# An Examination of Trunk and Right-Hand Coordination in Piano Performance: A Case Comparison of Three Pianists

**DOI:** 10.3389/fpsyg.2022.838554

**Published:** 2022-06-02

**Authors:** Craig Turner, Peter Visentin, Deanna Oye, Scott Rathwell, Gongbing Shan

**Affiliations:** ^1^Department of Kinesiology, University of Lethbridge, Lethbridge, AB, Canada; ^2^Department of Music, University of Lethbridge, Lethbridge, AB, Canada

**Keywords:** piano performance, movement coordination, anthropometric kinematics, skilled behavior, motion capture, motor learning and control

## Abstract

Playing the piano at expert levels typically involves significant levels of trial-and-error learning since the majority of practice occurs in isolation. To better optimize musical outcomes, pianists might be well served by emulating some of the practices found in sports, where motor learning strategies are grounded in biomechanics and ergonomics in order to improve performance and reduce risk of performance-related injuries. The purpose of the current study is to examine trunk-hand coordination and preparatory movement strategization in piano performance, while considering the influence of anthropometry, skill level of the performer, and musical context. Using a ten-camera motion capture system, movement of C7 and right-hand distal phalanges was tracked at three different playing speeds during performance of an excerpt from Beethoven’s “Appassionata” Sonata. There were three participants: two males and one female of differing anthropometric characteristics and skill levels. Motor strategization was examined. Expertise influenced starting trunk position: Initiation intervals and trunk range of motion (ROM) both suggested anthropometry to be a performance factor. For the shortest performer, trunk movement appeared to be used as an efficiency measure to compensate for a shorter arm reach. Skill level was revealed by examining right-hand velocity at the fastest tempo. The current study hypothesizes that an examination of proximal-to-distal preparatory strategies in terms of anthropometry and skill level can help to optimize motor learning for pianists. To realize piano performance as a whole-body skill and encourage healthy practice, pedagogy needs to educate learners regarding fundamental biomechanical and ergonomic principles, movement optimization, and movement strategization in the service of artful performance.

## Introduction

It is estimated that 17 million people worldwide play the piano at an advanced level (Harris, 2017). Attaining an advanced level of piano performance skills requires years of training. In western pedagogical traditions, music learning occurs in a one-on-one student–teacher setting. Typically, students receive only one instructional session per week and the majority of time spent learning involves individually motivated practice. For piano students enrolled at university, weekly practice hours can be as high as 39 h per week (Kaufman-Cohen et al., 2018). For professional pianists, average practice hours can range from 3.30–3.83 h per day (Jabusch et al., 2009; Moñino et al., 2017) or 13.70–27.00 h per week (Ericsson et al., 1993; Krampe and Ericsson, 1996; Allsop and Ackland, 2010). Given the long hours of necessary self-directed practice, the learner must be equipped with both cognitive and motor-based learning strategies that are grounded in deliberate and directed practice.

Motor learning research has shown that engaging in deliberate practice improves skill acquisition (Ericsson et al., 1993; Baker et al., 2020). With regard to the biomechanics of music performance and the ergonomics of interacting with a piano, deliberate practice can be a challenge because (a) most music teachers are not trained in the fundamentals of movement science, and (b) the majority of music biomechanics pedagogy is based in empirical methodologies—the subjective experience of the teacher (Visentin et al., 2008; Shan et al., 2013). Since there is a strong reliance on teachers’ abilities to communicate personal perceptions of their own experiences, this model of pedagogy has limitations (Turner et al., 2021a). Specifically, learning will tend to include a significant amount of trial-and-error practice. This can result in the acquisition of “bad practice habits” or the development of idiosyncratic playing styles, which has implications for increased risk of playing-related injuries (Fry, 1987; Guptill and Zaza, 2010; Moraes and Antunes, 2012; Turner et al., 2021b). To better optimize learning, students of piano need to be provided with motor learning strategies that are grounded in biomechanics and ergonomics. Unfortunately, in existing research, there is sparse discussion of meaningful motor learning strategies devoted to optimizing piano performance (Turner et al., 2021a).

In piano performance, a performer is required to physically move. A piano keyboard has 88 keys that are fixed in location and span a distance of 1.22 m. Since the keyboard is stationary, performers must adjust their position to the piano, coordinating trunk and upper limb movement according to the demands of the musical score. For high-level performers, movement seems autonomous. However, it is the product of long-term practicing involving deliberate decision-making and mental planning in the pursuit of specific musical goals (Bangert et al., 2014). From a biomechanical standpoint, utilization of the trunk during coordinated movement provides a more efficient means of executing motor skills (Turner et al., 2021a). In sports, the use of proximal musculature to facilitate distal movement is well documented (Shan and Westerhoff, 2005; Zhang and Shan, 2014; Zhang et al., 2016). In music, proximal-to-distal movement sequencing has been examined for drumming (Altenmüller et al., 2020) and piano keystrokes involving a “struck touch” (Furuya and Kinoshita, 2007; Verdugo et al., 2020). Pappa et al. (2020) reported that adolescent novice pianists exhibited more trunk and hand movement while playing scales at fast tempos compared to more experienced adolescent pianists. By analyzing the timing of shifts in balance during performance of a virtuosic piano composition, Turner et al. (2021a) concluded that movement coordination is dependent on timely preparation; when and how a pianist prepares for movement greatly affects performance. These findings support Godoy et al. (2010) and Godøy (2013) who identify the importance of movement preparation for musical co-articulation in terms of motor chunking and sound-producing actions. Given the complexity of both cognitive and biomechanical preparation, study of individualized factors helps us understand how and why preparatory strategies are executed during piano performance.

Optimizing performance strategies requires consideration of a pianist’s anthropometry and skill level in terms of the musical demands of the composition being performed. Anthropometrical characteristics dictate how a motor skill is learned (Ballreich, 1996). Because anthropometry differs among individuals, this suggests that most motor learning must be individualized. In music performance, motor behavior can vary greatly depending on the music being performed and the skill level of the performer. Advanced performers strategize and manipulate gross and fine motor skills in order to achieve artistic and interpretive musical outcomes (Shan et al., 2013). Mere repeatability is not the goal. This complexity makes the study of music performance and the application of motor learning methodologies very challenging.

In the current case study, two expert pianists and one intermediate pianist performed the last 9 measures of the 3rd movement in Beethoven’s Sonata in F Minor Op. 57 (“Appassionata”) at three different playing speeds. Preparatory movements involving timely coordination of the trunk and right hand (RH) were analyzed. The purpose of the current study is to examine trunk-hand coordination and preparatory movement strategization in piano performance, while considering the influence of anthropometry, skill level of the performer, and musical context.

## Materials and Methods

### Participants

Three pianists (two males and one female) of differing anthropometric characteristics and skill levels were recruited for the study (Table 1). Participants 1 (P1) and 2 (P2) were professionals with completed Doctorate degrees. Participant 3 (P3) was an intermediate-level pianist with 11 years of piano study. All participants were right-hand dominant and, according to the Beighton Hypermobility protocol, exhibited no signs of hypermobility. Participants gave written informed consent after a briefing on the research protocol and procedures, all of which were approved by the University of Lethbridge Human Subject Research Committee [approval #2018–098].

**Table 1:** Sex, select anthropometric measures, handedness, and experience level of each participant in the study.

Participants	Sex	Body height (m)	Hand span (m)	Hand length (m)	Forearm length (m)	Upper arm length (m)	Handedness	Experience level
P1	Female	1.645	0.178	0.172	0.253	0.289	Right	Expert
P2	Male	1.900	0.203	0.219	0.270	0.331	Right	Expert
P3	Male	1.735	0.193	0.192	0.258	0.326	Right	Intermediate

### Musical Excerpt

**Figure 1:**
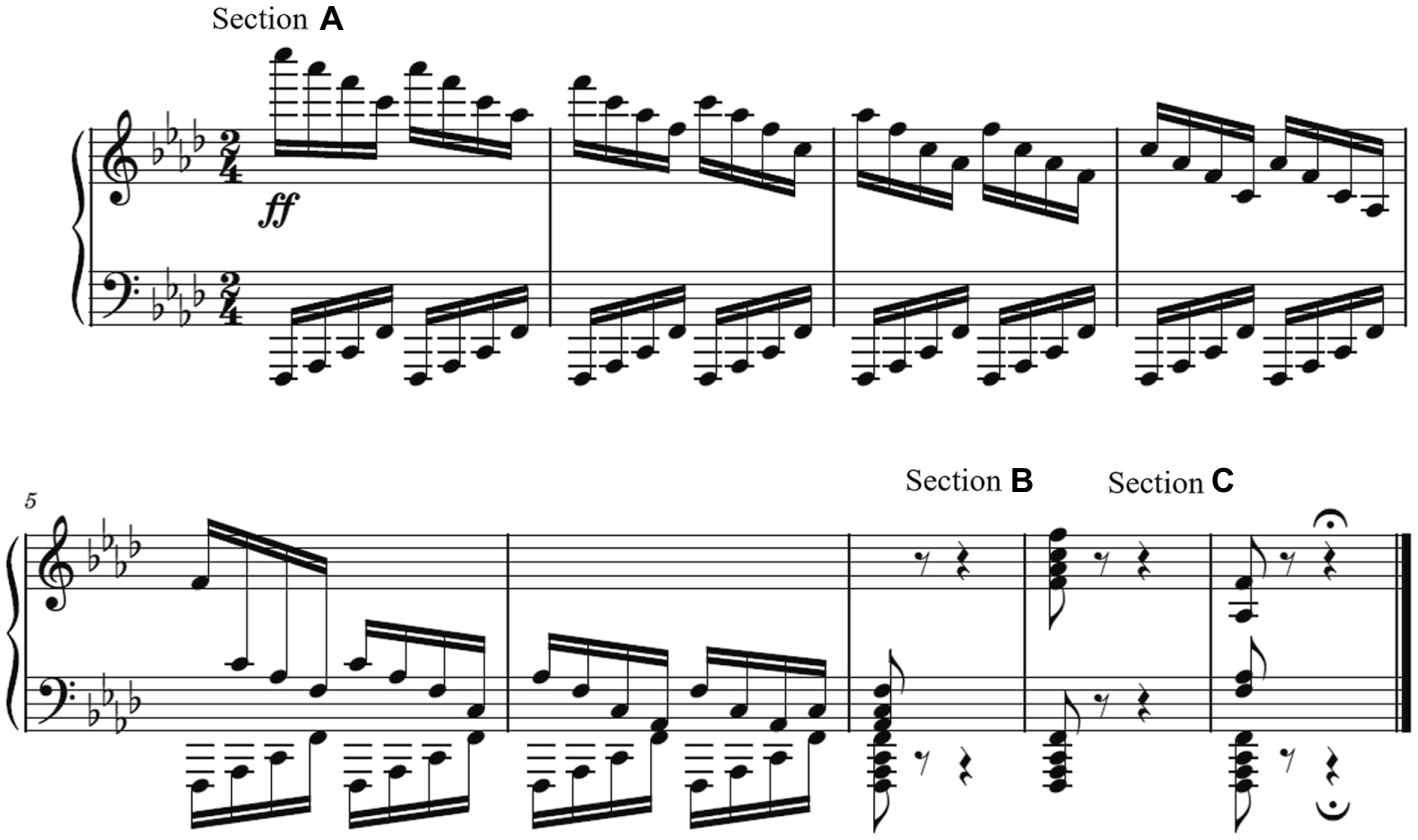
The last 9 measures (mm. 363–371) from the 3rd movement of Beethoven’s Sonata in F Minor Op. 57 (“Appassionata”) with three identified motor behavior phases: gradual RH descent (Section **A**), 2-octave medial-to-lateral RH jump (Section **B**), and 1 octave lateral-to-medial RH jump (Section **C**).

Participants performed the last 9 measures (mm. 363 to 371) from the 3rd movement of Beethoven’s Piano Sonata in F Minor Op. 57 (“Appassionata”; Figure 1), an excerpt exemplary of the virtuosic literature from the early Romantic period. Three playing speeds were examined: 6, 8, and 10 notes/s (N/s). Performing at the fastest tempo (10 N/s) is an expert task. By examining three different tempos, it was possible to evaluate motor strategization differences that might be due to tempo. Participants were instructed to perform in accordance with Beethoven’s instructions in the score but without using the pedals. Excluding the pedals permitted focus on upper body movement without the confounding variable of right-foot pedaling. Participants were given the music two weeks prior to data collection. Notwithstanding that there was no requirement to memorize the excerpt, based on visual observation, none of the participants read from the score during performance.

The musical excerpt divides into three sections (A, B, and C) based on distinct motor demands for the RH: A) a gradual, descending series of “broken” 4-note chords covering a lateral-to-medial distance of 57 cm (3.5 octaves), (B) a medial-to-lateral jump using “blocked” chords and covering a distance of 32.5 cm (2 octaves), and (C) a lateral-to-medial jump using “blocked” chords and covering a distance of 16.25 cm (1 octave). Throughout the excerpt, the LH was stable in terms of medial/lateral position, playing a repetitive 4-note pattern for the first six measures, and the same four notes in “blocked” chords for the last three measures. These kinds of motor behavior demands are common in western musical tradition. Many pedagogical sources, such as “Essential Finger Exercises for Obtaining a Sure Piano Technique” by Dohnányi (1929), deliberately cultivate medial–lateral motor behaviors using chordal patterns.

### Data Collection Procedure and Analysis

To quantify movement during performance, reflective markers were placed on six key anatomical landmarks and a ten-camera motion capture system (VICON MX40, Oxford, England) recorded positional and kinematic data. Capture frequency was 200 Hz (calibration error < 0.6 mm). One marker was placed on C7 and five were placed on the distal phalanges of the RH. The C7 marker provided a reference for trunk position while the RH position was determined using the five markers on the RH. 88 markers were placed on the keys of the piano to identify keystroke timing and accuracy.

**Figure 2:**
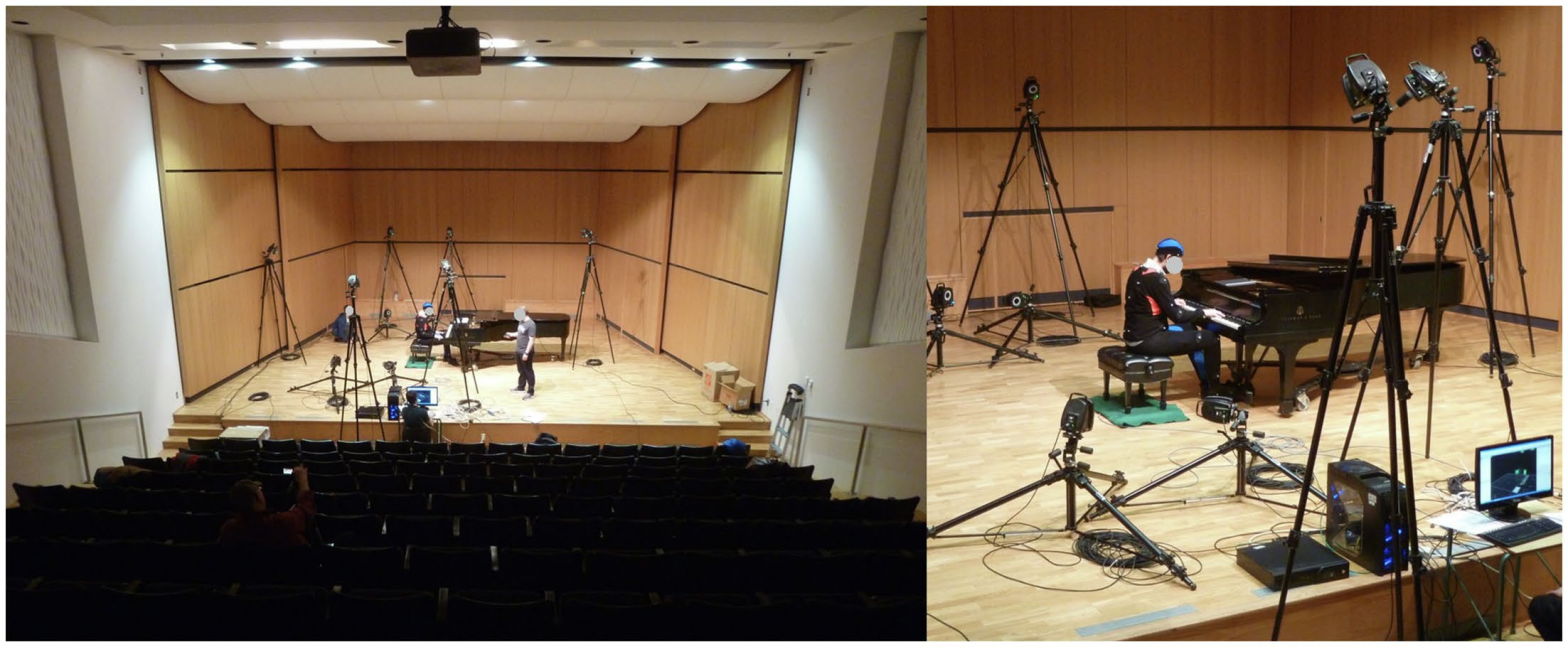
The experimental set-up of the motion capture system in the concert hall.

**Figure 3:**
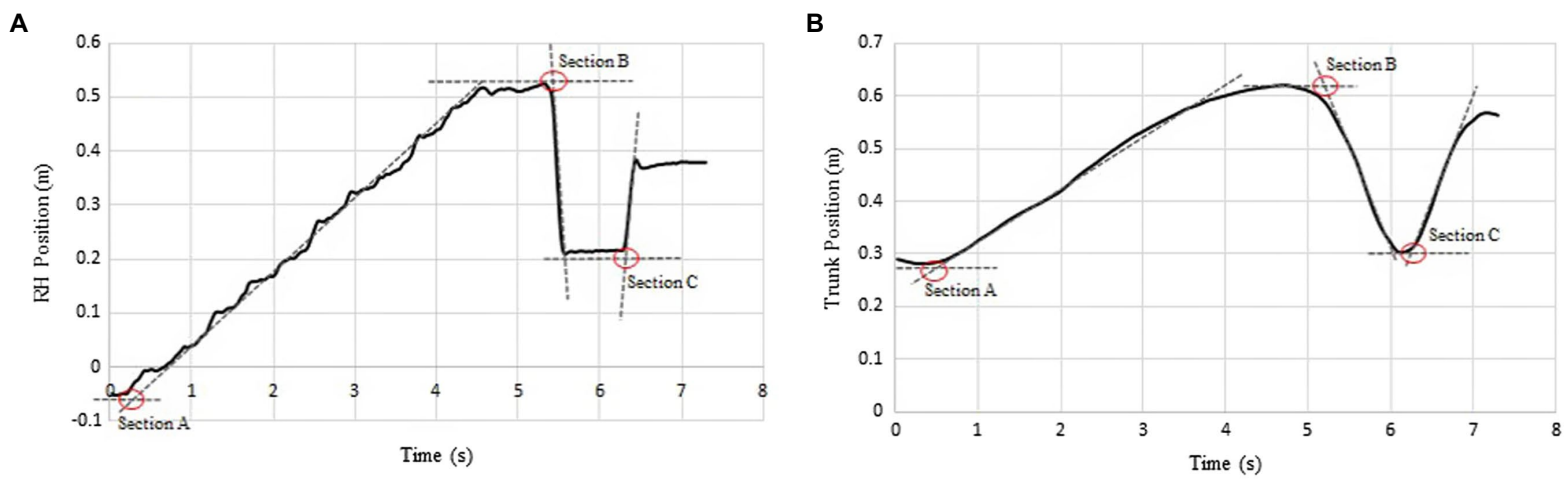
**(A,B)** An example of the method used to calculate initiation time points. The intersection points between the initial position and positional slope (red circles) indicate the initiation time for the RH and trunk in each section of the musical excerpt.

To simulate a realistic performance setting, participants performed on a 9-foot New York Steinway grand piano in a concert hall (Figure 2). A metronome was used to regulate tempo (playing speed), which made possible comparison between participants. Otherwise, participants were permitted interpretive latitude according to their own artistic understanding of the musical passage. Additionally, each pianist was permitted to adjust the piano bench height and position according to personal preference. By employing this kind of observational methodology, where movements are minimally controlled by experimental design (Anguera et al., 2017), unrealistic motor behavior strategies arising from the control of performance parameters were avoided (Turner et al., 2021a). To establish timing of motor control, medial/lateral movement for the RH and trunk was analyzed using a global center point for the RH and the C7 marker. The positional data of the five RH markers were averaged to determine a global center point for the RH. RH position is plotted in Figure 3. Medial/lateral movement of the trunk (C7) is plotted in Figure 3B. “Best fit” slopes for each of the graphs were determined (dotted lines—Figures 3A,B). The intersection of these slopes establishes time points where changes in motor control occur. Control changes coinciding with the beginnings of A, B, and C are identified using red circles (Figures 3A,B). Initiation intervals were defined as the difference in time between trunk and RH time points. Positive values indicate that the trunk starts moving before the RH and negative values indicate the converse. Since the LH remained in a constant register, playing the same four notes throughout the excerpt, it can be viewed as a stable biomechanical factor. For the current research, the RH was analyzed because of its greater activity.

## Results

Medial–lateral starting position of the trunk differed among participants. The two expert performers began with a C7 starting position that favored the RH. For P1, C7 was, on average, 65.9 mm closer to the RH than to the LH and for P2, this distance was 33.9 mm. For the intermediate performer, C7 starting position favored the LH by 7.2 mm.

Initiation intervals for each participant are shown in Table 2. During the first section of music (A), interval values are negative, indicating that all participants initiated with the RH. As tempo increased, initiation times for P1 decreased while, for P2 and P3, initiation times increased from 6 N/s to 8 N/s and decreased at 10 N/s (Table 2). Average initiation times were distinctly different for each subject, −0.450 s, −0.927 s, and − 0.668 s for P1, P2, and P3, respectively. During section B, trunk movement preceded RH movement. For the expert pianists, P1’s initiation intervals were remarkably consistent across all tempos (0.205 s, 0.215 s, 0.185 s) while P2’s were more variable (0.350 s, 0.490 s, 0.260 s). For P3, the intermediate-level pianist, initiation intervals steadily decreased as tempo increased (0.355 s, 0.210 s, 0.050 s). During section C, P1’s initiation intervals were consistently close to zero (−0.050 s, 0.015 s, −0.005 s), with an average of −0.013 s across all tempos. For P2 and P3, initiation intervals decreased as tempo increased (0.695 s, 0.220 s, −0.155 s) and (0.445 s, 0.155 s, 0.195 s) for P2 and P3, respectively.

**Table 2:** The initiation intervals between the trunk and RH for all three sections of the music across the three tempos. Trunk initiations are in bold print.

Participants	Tempo (N/s)	Music Sections					
		A (s)	Average of A (s)	B (s)	Average of B (s)	C (s)	Average of C (s)
P1	6	−0.885	−0.450 ± 0.385	**0.205**	0.202 ± 0.015	−0.050	−0.013 ± 0.033
	8	−0.330		**0.215**		0.015	
	10	−0.145		**0.185**		−0.005	
P2	6	−1.080	−0.927 ± 0.522	**0.350**	0.367 ± 0.116	**0.695**	0.253 ± 0.426
	8	−1.355		**0.490**		**0.220**	
	10	−0.345		**0.260**		−0.155	
P3	6	−0.465	−0.668 ± 0.179	**0.355**	0.205 ± 0.153	**0.445**	0.265 ± 0.157
	8	−0.800		**0.210**		**0.155**	
	10	−0.740		0.050		**0.195**	

Trunk range of motion (ROM) across all tempos are shown in Table 3. For each musical section (A, B, and C), P1 had the largest trunk ROM, P2 had the smallest trunk ROM, and P3’s ROM was somewhere in between. Across musical sections A, B, and C, each of the participants had highest trunk ROM in section A and lowest in section C, with section B ROM falling in between. Looking at the extreme speeds, slowest and fastest tempos only: in section A, ROM for P2 increased while it decreased for P1 and P3; in section B; ROM increased for P1 and P2 while it decreased for P3; and, in section C, ROM increased for P1 while it decreased for P2 and P3.

**Table 3:** Trunk ROM (mm) across all tempos and sections of the musical excerpt.

Trunk range of motion (mm)												
	Section A				Section B				Section C			
Participants	6 N/s	8 N/s	10 N/s	Average	6 N/s	8 N/s	10 N/s	Average	6 N/s	8 N/s	10 N/s	Average
P1	313.3	340.0	272.2	308.5	262.1	318.5	270.1	283.6	191.0	265.6	254.2	237.0
P2	147.4	155.7	198.8	167.3	76.3	80.4	94.8	83.8	66.2	55.8	32.3	51.4
P3	188.0	167.7	174.0	176.6	164.5	135.5	107.3	135.8	83.8	89.0	27.3	66.7

**Figure 4:**
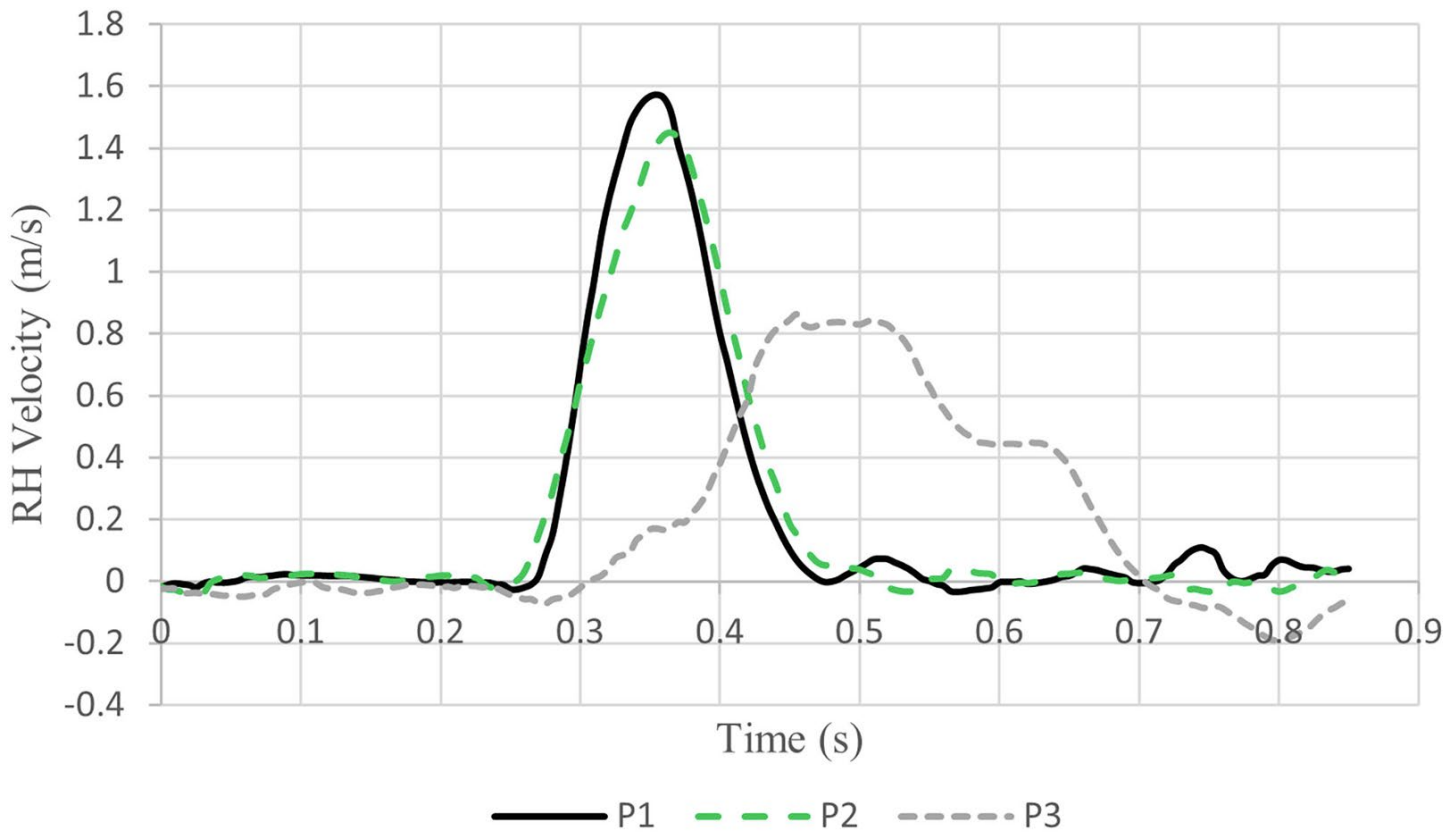
RH velocity for all pianists during section C at 10 N/s.

In Figure 4, medial–lateral RH velocity of the two expert pianists (P1 and P2) showed similar motor behaviors while the intermediate performer (P3) had a markedly different motor behavior. Maximum RH velocities achieved by P1 and P2 were 1.6 m/s and 1.4 m/s. For P3, maximum RH velocity was 0.8 m/s. Velocity curve contours showed similar differences. For P1 and P2, velocity curve contours were smooth and continuous with medial–lateral movement completion requiring ~0.22 s while, for P3, the curve contour was irregular and movement completion required more than 0.5 s.

## Discussion

When performing compositions, such as Beethoven’s Appassionata Sonata, both proximal (trunk) and distal (hands) body structures must move; the distances between the notes are simply too big for the trunk to be static. When coordinating body segments for complex movements, relevant questions for musicians could be “what should move first?” and “how should it move?” By examining musical demands, ranges of motion in the trunk and the hands, initiation intervals, anthropometry, and expertise levels, the current study provides a means to examine these questions in a way that can be meaningful to musicians.

### Starting Position

Whether consciously rationalized or not, a pianist’s starting position is the first preparatory decision they must make. Position influences the availability of movement options for the performer, and consequently can be considered in terms of both expertise and anthropometry (Ackermann and Adams, 2003; Vantorre et al., 2014; James, 2018). In the current study, medial–lateral motor demands placed on the RH were considerably greater than those for the LH. Expert performers positioned themselves closer to the RH than the LH, effectively adjusting skeletal alignment to provide better proximal support for the RH. In contrast, the intermediate performer’s starting position, equidistant between his hands, fails to recognize the demands imposed upon the RH, a finding that is likely linked to his lower skill level. Given that there is a relationship between preparatory movements and sound production, the intermediate performer’s lack of preparation may be a consequence of a still developing perceptual schema of body awareness (Godøy, 2011). The two expert players, because of greater body awareness developed through long-term training, “naturally” positioned themselves asymmetrically to favor the hand executing the more difficult passage work.

In addition to expertise, anthropometry underpins data regarding starting position. P1, the shortest performer, started 65.9 mm closer to the RH than to the LH. For P2, the tallest performer, this positional asymmetry was only 33.9 mm. Simply, P1’s shorter reach required greater compensation from the trunk as a means of supporting fine motor execution in the RH. For P3, given his stature, it seems reasonable to expect that playing optimization would have required him to position his trunk somewhere between 33.9 mm and 65.9 mm closer to the RH. The current paper asserts that a learning environment sensitive to biomechanics and the influence of anthropometry could help such a performer improve ease of playing and performance outcomes.

### Initiation Intervals

Section A of the music has continuous playing involving movement throughout the right limb. Since the right limb must begin in an extended position, the consistent initiation of movement with the RH by all participants can be understood from a standpoint of effort minimization. The RH has a greater medial–lateral mobility than the trunk and, since it has less mass, it is the easier segment to move. This underpins the role of the RH as the initiator of movement in section A of the excerpt. Average initiation intervals were smallest, largest, and in between for P1, P2, and P3, respectively (Table 2). The small initiation interval for P1 indicates that she is moving the trunk more closely in tandem with the RH. In using this strategy, proximal-to-distal skeletal support is better maintained throughout the entire passage. For P2, longer upper limbs permitted a greater medial–lateral right arm reach and a correspondingly smaller reliance on trunk movement. His larger initiation interval, nearly double that of P1, indicates that he was less dependent on moving the trunk to optimize skeletal alignment in support of the RH. For P3, initiation times fell in between that of P1 and P2 suggesting his stature, intermediate to P1 and P2, to be a determining factor.

In music section B, the presence of “rests” in the music influences the motor strategization. During rests, a pianist does not play any notes, so the body has greater behavioral freedom in preparation for upcoming playing demands. In spite of this increased freedom, in section B, all participants initiated movement from the trunk. This supports the idea expressed by Furuya and Altenmüller (2013) that proximal-to-distal motor coordination might help optimize motor behavior. This furthers the notion that musical rests may present pianists with opportunity to prepare and optimize motor strategies.

In section B, other musical demands also affect movement; a *fortissimo* (“very loud”) “blocked” chord occurred after the RH medial-to-lateral jump of 32.5 cm (two octaves), making trunk movement necessary to support the RH. This substantiates the findings of Verdugo et al. (2020) with regard to the involvement of the trunk during *forte* (“loud”) playing. For P1, initiation intervals across all tempos were remarkably consistent (Table 2), with less than 3/100ths of a second difference between trials. This consistency suggests P1’s expertise to be very high. For P2 and P3, initiation intervals varied somewhat more than for P1 (0.367 s ± 0.116 and 0.205 s ± 0.153 for P2 and P3, respectively). Differences observed for P2 and P3 are small, also showing high levels of expertise.

Section C of the music ends the composition and requires a “very loud” finishing gesture. P1’s use of simultaneous trunk and RH movement (initiation intervals across all tempos varied less than 0.065 s), show her to be maximizing trunk support to achieve this effect. For P2 and P3, the RH became more closely synchronized with the trunk at the two faster tempos. While P1 employed this strategy at all tempi, P2 and P3 selectively optimized trunk support for the RH only as the difficulty of playing increased because of increased speed. From this, it appears that Beethoven’s “very loud” chordal finishing gesture is enhanced by greater synchronization between trunk and hand movement at faster tempos.

### Trunk ROM

In the current study, average trunk ROM was linked to anthropometry. Average ROM was largest for P1 (shortest performer), smallest for P2 (tallest performer), and somewhere in between for P3 (medium-sized performer; Table 3). This suggests anthropometry to be an important factor for trunk movement. For P1, the participant with the shortest arm reach, a larger trunk ROM may have served as a compensatory mechanism to optimize RH support. P2’s longer upper limbs permitted a greater medial–lateral arm reach and greater leverage when striking the piano keys, so less trunk movement was required in general. For P3, trunk ROM was intermediate to those of P1 and P2, a finding consistent with his physical stature. Comparing sections of music, each participant’s average trunk ROM was greatest in A, less in B, and least in C. This phenomena appears to be coupled with RH playing demands; the RH moves furthest in A, less in B, and least in C.

Tempo-dependent trunk ROM is revealing with regard to movement strategization. P1 used much more trunk ROM than either P2 or P3. For P1, reducing proximal movement trunk ROM had utility as an efficiency measure. At faster tempos, since there was less time to move, P1 reduced trunk movement (Table 3). For P2 and P3, because they were moving so much less than P1 overall, there was little efficiency gain to be had by employing P1’s motor strategy.

### Right Limb Coordination

Analyzing RH coordination provides insight on expertise. The complex RH movement patterns of P3 are in stark contrast to those of P1 and P2. This can be clearly seen in Figure 4, which compares RH velocities of all participants for section C of the music at 10 N/s. P3 moves in “fits and starts” while P1 and P2 have smooth and continuous RH velocity changes. For P3, trunk movement is nearly frozen in this trial, moving only 27.3 mm, which is evidence of his lesser expertise; his RH must attempt to compensate for reduced trunk movement. This reinforces the assertion of Drake and Palmer (2000) that expert pianists anticipate during performance than less experienced pianists.

In the context of piano performance, movement strategies must be sublimated into artistic intention, and these are expertise-dependent. A further consequence of suboptimal movement behaviors is that they have the potential to increase risk of playing-related injury (Taimela et al., 1990; Turner et al., 2021b). In the case of P3, pedagogical instruction directing his attention toward trunk movement may have improved his coordination.

### Limitations and Future Work

The current study asserts that anthropometry, skill-level, and musical context are all necessary considerations when learning to perform the piano. As a case comparison, it only involves three participants, two experts, and one intermediate-level participant. As such, the utility of the current study lies in the rationalized discussion of the data. Because of the relatively stable positioning of the LH, only the RH was examined in the current study. Since piano performance involves an array of musical contexts, more studies are needed to come to a complete understanding of how musical context directs motor strategization; a larger body of work examining practical biomechanical strategies is needed. Lastly, in the current study, the authors were careful to make the experimental process as natural as possible for the performers, so that they would not deliberately manipulate their “normal” playing style to accommodate experimental design. Notwithstanding this, the data gathering process was not a “normal” situation for the performers and the possibility of some subtle playing alterations cannot be eliminated. We propose that a line of research which considers cause and effect of the conscious manipulation of movement strategies in the service of artistic performance could have broad ranging application by employing systematic observational methodology that utilizes polar coordinate or T-pattern analysis to detect movement regularities among pianists.

## Conclusion

In the current case study, three pianists’ timely coordination of the trunk and right-hand (RH) preparatory movements were analyzed during performance of the last 9 measures of the 3rd movement in Beethoven’s “Appassionata” Sonata, at three different playing speeds. The musical excerpt had three different sections requiring distinct motor behaviors for the RH, while the LH remained relatively stable. This permitted examination of preparatory strategies for each of the various motor behaviors. Starting position, initiation intervals, trunk ROM, and right limb coordination were analyzed. Despite similar initiation strategies for all pianists (distal-to-proximal in section A and proximal-to-distal in sections B and C), the trunk appeared to 1) move within the RH medial–lateral confines of each musical section (musical context) and 2) be a source of compensation for anthropometry, specifically for P1, the shortest pianist. Evidence of level of expertise was found in the positional preparation relative to the keyboard and RH coordination at the fastest speed.

The current study underpins the utility of recognizing the influence of biomechanics, anthropometry, skill level, and motor learning strategies in piano learning and performance. Comparative analyses, such as those of the current study, provide biomechanical and ergonomic perspectives that have potential to optimize the process of learning to play the piano. Individualization of preparatory strategies should be recognized in terms of the anthropometry of the learner. The generally accepted concept that proximal body structures initiate movement may not be possible depending on musical context. In this case, expertise and anthropometry need to be considered more carefully. In addition to improving musical outcomes, this has the potential to optimize movement intentionality in the service of injury prevention. Future studies can contribute more effectively to motor learning by focusing on the questions “how does the musical context require that a motor strategy be employed?” and “what motor strategization should be used?” These two questions are eventually faced by every musician and need to underpin the motivation of music biomechanics research that is intended to be cross-disciplinary.

## Data Availability Statement

The datasets presented in this article are not readily available because as a case comparison study, it may make subjects identifiable to third parties. Requests to access the datasets should be directed to g.shan@uleth.ca.

## Ethics Statement

The studies involving human participants were reviewed and approved by the University of Lethbridge Human Subject Research Committee. The patients/participants provided their written informed consent to participate in this study.

## Author Contributions

CT, PV, and GS contributed to all facets of the research (concept, design, data collection, analysis, interpretation, and manuscript preparation). DO contributed to the design of the research protocol and data interpretation. SR contributed to the revisions of the manuscript. All authors contributed to the article and approved the submitted version.

## Funding

This research was supported by Discovery Development Grant of National Sciences and Engineering Research Council of Canada (NSERC) (DDG-2021-00021).

## Conflict of Interest

The authors declare that the research was conducted in the absence of any commercial or financial relationships that could be construed as a potential conflict of interest.

## Publisher’s Note

All claims expressed in this article are solely those of the authors and do not necessarily represent those of their affiliated organizations, or those of the publisher, the editors and the reviewers. Any product that may be evaluated in this article, or claim that may be made by its manufacturer, is not guaranteed or endorsed by the publisher.
